# One-stage Surgery for Intracardiac Leiomyomatosis

**DOI:** 10.21470/1678-9741-2023-0291

**Published:** 2025-02-11

**Authors:** Xin Luo, Xin Wen, Yiyuan Li, Jichun Zhao, Xiyang Chen, Qiang Guo, Bin Huang

**Affiliations:** 1 Department of Vascular Surgery, West China Hospital of Sichuan University, Chengdu, Sichuan, People’s Republic of China

**Keywords:** Cardiopulmonary Bypass, Chambers, Transesophageal Echocardiography, Leiomyomatosis, Sternotomy, Surveillance in Disasters, Thrombectomy

## Abstract

**Introduction:**

Intracardiac leiomyomatosis is a rare, histologically benign, but biologically aggressive tumor developed from uterus. This study aimed to summarize our experience with one-stage surgery for intracardiac leiomyomatosis.

**Methods:**

We retrospectively reviewed seven patients who underwent surgical treatment for intracardiac leiomyomatosis between May 2016 and November 2021.

**Results:**

All seven patients were female, aged 35 to 57 years. All lesions in the veins and cardiac chambers were removed entirely. Four of the seven patients received tumor thrombectomy through an abdominal approach. The other three patients received median sternotomy and cardiopulmonary bypass. No perioperative deaths or serious complications occurred during the observation period. The mean operation time in the abdominal approach group was shorter than that in the cardiopulmonary bypass group (308.9 ± 93.2 minutes vs. 486.3 ± 108.6 minutes; P=0.031). Blood loss during surgery in the abdominal approach group was less than that in the cardiopulmonary bypass group (1625 ± 216 mL vs. 2500 ± 1080 mL; P=0.148). All seven patients were free from tumor recurrence or death during the follow-up.

**Conclusion:**

For patients with intracardiac intravenous leiomyomatosis single-stage operation through an abdominal approach under the surveillance of intraoperative transesophageal echocardiography without the need for cardiopulmonary bypass for specified patients is feasible. Patients in the abdominal approach group can benefit from a shorter operation time and less blood loss. In our small series of varied presentations and tumor extent, we have been able to avoid two-stage surgery, because even short-term interval between the two operations may result in recurrence.

## INTRODUCTION

**Table:** 

Abbreviations, Acronyms & Symbols	
BIIV	= Bilateral internal iliac vein
CECT	= Contrast-enhanced computed tomography
CPB	= Cardiopulmonary bypass
CTV	= Computer tomography venography
ICL	= Intracardiac leiomyomatosis
ICU	= Intensive care unit
IVC	= Inferior vena cava
IVL	= Intravenous leiomyomatosis
LIIV	= Left internal iliac vein
LMWH	= Low-molecular-weight heparin
PTFE	= Polytetrafluoroethylene
RA	= Right atrium
RIIV	= Right internal iliac vein
ROV	= Right ovarian vein
TEE	= Transesophageal echocardiography

Intravenous leiomyomatosis (IVL) is an intravascular proliferation of smooth muscle cells from the uterus^[1]^. IVL is a rare, histologically benign, but biologically aggressive tumor, and intracardiac leiomyomatosis (ICL) refers to the tumor that involves the cardiac chamber through venous channels^[2-4]^. Patients may be asymptomatic despite extensive iliac vein extension, and presentations vary greatly and can be associated with either gynecologic symptoms, such as pelvic pain and menstrual alteration, or symptoms secondary to direct cardiac involvement, like right heart failure or syncope^[5,6]^. Due to the fact that the tumor is hormonally responsive, antiprogesterone and antiestrogen therapy may potentially be beneficial in controlling the tumor progression, and surgical resection remains the mainstay of curative treatment for patients with ICL^[7]^. The first total resection of an ICL was reported by Ariza et al.^[8]^ in 1982 with a delayed laparotomy after resection of the intracardiac tumor. Subsequent reports in the literature described either two-stage surgery involving resection of the intravenous and intracardiac lesions at two different times or a one-stage surgery that takes advantage of cardiopulmonary bypass (CPB) or hypothermic circulatory arrest to allow excision of all lesions at once. Although various ways of surgical management for ICL were reported, the optimal surgical approach remains unclear. To the best of our knowledge, no more than 200 ICL cases have been reported until now. Here, we present seven cases of ICL and outline our experience in their surgical treatment.

## METHODS

### Patients

Medical records of seven patients who underwent surgical treatment for ICL between May 2016 and November 2021 at West China Hospital, Sichuan University, were retrospectively reviewed. This study was approved by the ethics committees of West China Hospital, Sichuan University (20211018). Informed consent was signed by each patient.

### Operative Technique

**Fig. 1:**
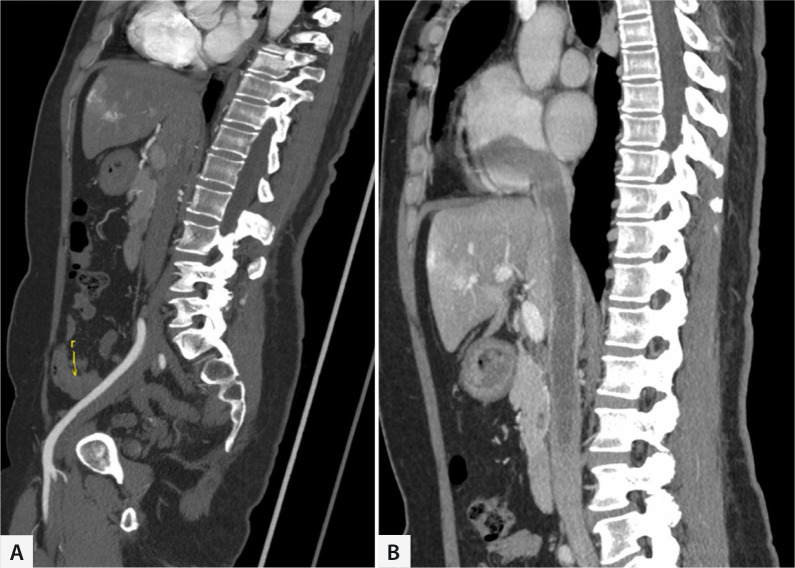
Preoperative computed tomography images of intracardiac leiomyoma. A) Lesion in iliac vein. B) Lesion in inferior vena cava and cardiac chamber.

Anatomical structure of the tumor was detected by preoperative contrast-enhanced computed tomography (CECT). When the width of the post-hepatic inferior vena cava (IVC) was larger than the maximum diameter of the mass inside the right atrium (RA) and there were no adhesions between the lesions and cardiovascular intima, a single-stage surgery was performed without the need for median sternotomy, CPB graft, or hypothermic arrest; in order to ensure an adequate surgical working space and optimal exposure of the lesion, we utilized a midline abdominal incision extending from the xiphoid process to the pubic symphysis. Routine intraoperative transesophageal echocardiography (TEE) was performed to confirm the tumor's detachment and floatation inside the IVC and RA. Through the abdominal approach, we pushed the intestines to the left side of the abdominal cavity, then opened the right colonic gutter, placed wet gauze under the liver, and carefully lifted the liver using an abdominal retractor; segments from post-hepatic IVC to iliac veins were exposed, and bilateral gonadal veins, uterus, and bilateral ovaries were also exposed if necessary; surgical procedures may lead to the displacement of the tumor, so TEE was employed throughout the entire operation to closely monitor whether the lesion exhibited any movement towards the proximal end. As shown in Figure 1A, one blocking band was used to encircle the post-hepatic IVC, two blocking bands were used to encircle the bilateral renal veins, and two blocking bands were used to encircle the infrarenal IVC at 5-cm intervals. After unfractionated heparin were delivered intravenously, a longitudinal incision was made between the blocking bands at infrarenal IVC. The upper tumor was then pulled through the incision, post-hepatic IVC and infrarenal IVC were gradually controlled by pulling blocking bands upwards to tighten vascular wall. When the tumor was dissected and TEE confirmed that there was no residual or detachment of the upper segment tumor, we blocked the IVC at the edge of the incision by tightening blocking bands completely. Pelvic mass was subsequently resected by the gynecologist, the rest of the tumor in the IVC and the iliac vein was pulled through the incision, using 5-0 Prolene® with an "outside-in, outside-out" continuous suturing strategy to suture the incision. In case of IVC or iliac vein reconstruction, non-ringed polytetrafluoroethylene (PTFE) or ringed PTFE grafts were the conduits of choice.

For patients with the width of the post-hepatic IVC smaller than the maximum diameter of the mass inside the RA or adhesions between the tumor and cardiovascular intima, CPB via the cannulas in the ascending aorta and superior vena cava with cardioplegic cardiac arrest or not was placed on. In this type of surgery, firstly, the tumor was transected through venotomy within the abdomen. Then, the tumor in the cardiac chambers and the IVC above the incision was removed through a right atriotomy, and the remaining part of the tumor was removed similarly to the aforementioned surgery. If the tumor was densely adherent to the tricuspid valve, the valve was repaired or replaced. Postoperatively, patients were treated with low-molecular-weight heparin (LMWH) during the hospital stay and rivaroxaban after discharge for 3-6 months.

### Data Collection

Patients’ demographics, clinical presentations, preoperative work-up, surgical findings, morbidity, and follow-up data on survival and recurrence were obtained. Specifically, the basic information, including patients’ age, symptoms, and clinical features, was reviewed. Before surgery, patients underwent a CECT scan of the chest, abdomen, and pelvis. The data included the IVC width behind the liver and the maximum diameter of the mass inside the RA measured on CECT. The surgical and anesthetic records were reviewed for the operation time, operative blood loss, and intraoperative blood transfusion. All patients were reviewed at 30 days to assess for complications using the Clavien-Dindo Classification^[9]^. Hospital stay after surgeries, intensive care unit (ICU) stays, and fatal events were recorded. Patients were followed at three, six, and 12 months after surgery, then annually afterward. An ultrasound examination of the IVC and iliac veins was performed at each follow-up visit, and a CECT scan was performed if abnormal findings were observed. The interval to local recurrence was defined as the time from initial surgery to radiological confirmation of local recurrence. Overall survival was measured from the time of the first operation to either death or the last follow-up.

### Statistical Analysis

Statistical analysis was performed using IBM Corp. Released 2011, IBM SPSS Statistics for Windows, version 20.0, Armonk, NY: IBM Corp., and all the data are presented as the mean ± standard deviation. An independent sample *t*-test was used to analyze and compare the perioperative data between the two groups. Two-sided *P*<0.05 was considered statistically significant.

## RESULTS

A total of seven patients were included in this study. As shown in Table 1, all seven patients were female, aged 35 to 57 years. Two of these patients were menopausal at admission. Six patients had symptoms, including chest tightness, syncope, dysmenorrhea, and edema of the lower limbs. Only one patient was asymptomatic. The right internal iliac vein was the most frequent path where the tumor entered IVC (three cases), followed by the right ovarian vein (two cases). Six patients had a history of hysteromyoma resection. Two patients previously received sternotomy with tumor extraction through a right atriotomy. The intervals between the first admission and the second admission of two patients were two and three months, respectively, moreover, both patients had tumor recurrence detected in the RA. Of these seven patients, six had tumors extending into the RA, as shown in Figure 1, and one had a tumor extending into the pulmonary artery. The maximum width of the IVC was 15.5 ± 4.2 mm, and the diameter of the tumor inside the RA was 15.3 ± 7.4 mm.

**Table 1:** Clinical characteristics and prognosis of seven patients with intracardiac leiomyomatosis.

Patient	Age (years)	Menopause	History of hysteromyoma surgery	Original vein	Symptoms	Follow-up (months)	Recurrence
1	57	Yes	Yes	RIIV	Chest tightness, edema of lower limbs	36	No
2	47	No	Yes	ROV	Syncope	73	No
3	50	No	Yes	ROV	None	52	No
4	49	No	Yes	RIIV	Syncope	59	No
5	51	Yes	Yes	LIIV	Syncope	10	No
6	35	No	No	BIIV	Dysmenorrhea	26	No
7	47	No	Yes	RIIV	Dysmenorrhea	6	No

BIIV=bilateral internal iliac vein; LIIV=left internal iliac vein; RIIV=right internal iliac vein; ROV=right ovarian vein

**Fig. 2:**
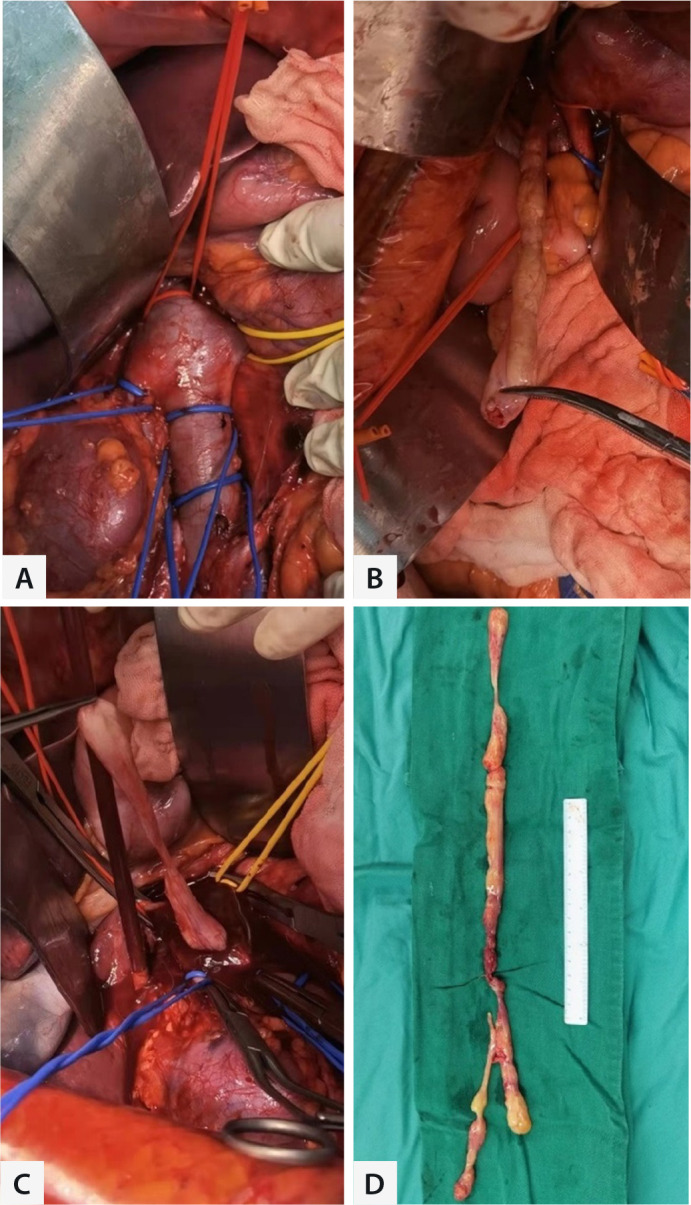
Surgical details display. A) Controlling the inferior vena cava (IVC) at the renal vein level. B and C) Removing the lesion from IVC. D) The whole leiomyoma.

All seven patients underwent one-stage surgery successfully, and the involved lesions in the veins and cardiac chambers were completely removed, as shown in Figure 2. Four of the seven patients received tumor thrombectomy through an abdominal approach, and the other three patients were treated with median sternotomy and CPB. For the patient with the invasion of the right ventricle and pulmonary artery, CPB-assisted surgery was performed under the condition of cardiac arrest. Adhesion was found between the mass and the tricuspid valve and could not be completely separated. Then, the tricuspid valve was completely removed and replaced with a bioprosthetic valve. In another patient, part of the lesion was adhered to and fused with the right external iliac vein. Hence, the right external iliac vein was removed and reconstructed with a ringed PTFE graft.

No perioperative deaths or serious complications occurred during the observation period. The average blood loss of the seven patients during the operation was 2000 ml, the average ICU stay was two days, and the average total length of stay after surgery was ten days. The mean operation time in the abdominal approach group was 308.9 minutes, shorter than that in the CPB group (308.9 ± 93.2 minutes *vs.* 486.3 ± 108.6 minutes; *P*=0.031). The blood loss during surgery in the abdominal approach group was less than that in the CPB group (1625 ± 216 mL *vs.* 2500 ± 1080 mL; *P*=0.148).

The follow-up period ranged from six months to six years, with a mean of 37 months. According to their ultrasound and computer tomography venography (CTV) images, all seven patients were free of tumor recurrence during the follow-up. All seven patients were alive at the last follow-up.

## DISCUSSION

Given its low incidence, there is still a lack of ICL management guidelines^[10]^. This study reported seven cases of ICL treated with different surgical approaches. We intend to share our experience and provide valuable guidance in treating this rare disease. In this study, single-stage operations through an abdominal approach without the need for median sternotomy in specified patients achieved promising results. The results of this study also demonstrated the importance of intraoperative TEE and the disadvantage of the two-stage surgery.

Complete surgical resection is still the gold-standard treatment for ICL, and it is the key to prevent recurrence. However, there is still some controversy about whether a one-stage or two-stage surgery should be applied^[7]^. For two-stage surgery, patients were treated with two separate operations, with the cardiac procedure performed firstly^[11]^, then the tumor was extracted from the IVC to the greatest extent possibly, and a second operation was performed via laparotomy later. This procedure can be performed in almost any type of ICL, especially in patients who are in poor condition^[12]^. However, two-stage surgery has a longer total operation time, more intraoperative blood loss, and more extended postoperative hospital stay^[13]^. According to a previous review, 32 articles provided a total sample of 110 cases^[7]^, the overall recurrence rate was 5.1%. In the abovementioned review, the intervals between two surgeries varied from seven days to two years, which resulted in a higher risk of tumor recurrence than that of a single-stage surgery. In our study, two patients previously received sternotomy with tumor extraction through a right atriotomy. However, the two patients experienced tumor recurrence shortly after the first surgery, which was a waste of medical resources and a heavy burden on the patients' economy and health.

Harris et al.^[14]^ first described a patient treated with a new approach involving a single-stage operation without the need for median sternotomy, CPB graft, or hypothermic arrest by resecting the tumor from the point of attachment in the abdominal portion of the IVC. This procedure is less invasive and can reduce operation time and hospital stay. However, the potential defect is that this strategy cannot control the distal end of the tumor intraoperatively, and if the tumor is fractured, acute pulmonary embolism and even death could happen during this procedure. Consequently, Li et al.^[10]^ and Xu et al.^[15]^ also reported their experience of abdominal approach with no CPB, and no intraoperative pulmonary embolism occurred under the surveillance of TEE. Li et al.^[10]^ introduced an anatomy-based guideline for four types of surgical strategies. Generally, our surgical strategy was similar to these strategies, and our results confirmed that, for specified patients, the perioperative outcomes of the abdominal approach are not inferior to traditional open-heart surgery for ICL, and less traumatic.

TEE is an ultrasonography-based cardiac imaging tool used in cardiac surgical procedures to facilitate informed surgical decision-making and manage intraoperative complications^[16]^. By intraoperative TEE, the tumor's mobility inside the RA and IVC can be observed. Besides, intraoperative TEE also helps to confirm the location of the proximal site of cancer during tumor thrombectomy through an abdominal approach. In this study, all seven patients received intraoperative TEE, and for the patient with the invasion of the right ventricle and pulmonary artery, adhesion was found by TEE between the mass and the tricuspid valve. Thus, monitoring with intraoperative TEE is highly recommended to guide decision making and avoid intraoperative pulmonary embolism.

The recommendation for postoperative antiestrogen therapy, tamoxifen or letrozole, remains controversial^[7]^. In some reports, hormonal therapies effectively prevented recurrence after incomplete resections^[17]^. However, the side effects of antiestrogen therapy, including osteoporosis and heart disease, cannot be ignored^[18]^. Few studies have been conducted to investigate the molecular and immunohistochemical profile of IVL^[19,20]^. A study assessed the clinical and morphological characteristics of 28 IVL and their correlation with molecular features and protein expression^[19]^. Results showed that four of the five IVLs associated with recurrence had a vascular morphology, and three had genetic alterations of 8q. Further studies are needed to assess clinical and morphological characteristics to identify biomarkers or therapeutic targets for IVL with aggressive clinical behavior.

Due to the fact that the surgery involves a long segment of veins and a period of bed rest postoperatively, there is a higher risk of venous thrombosis, especially deep vein thrombosis in the lower limbs. We typically start subcutaneous injection of LMWH for anticoagulation as early as the first day postoperatively, and upon discharge, transitioning to rivaroxaban for anticoagulation is advised. Monitoring of coagulation function and dosage adjustment is necessary during the anticoagulation period. Pneumatic compression devices for the lower limbs are recommended during the bed rest period to prevent deep vein thrombosis. Once the general condition improves, it is encouraged for the patient to engage in timely ambulation.

### Limitations

The retrospective character of this research was its most significant limitation. The number of cases was small because of the low incidence of IVL. Besides, there is a lack of data on long-term follow-ups after less invasive operations. More case data and longer follow-ups are needed to support the results presented here.

## CONCLUSION

For patients with intracardiac IVL in our center, single-stage operation through an abdominal approach under the surveillance of intraoperative TEE without the need for CPB for specified patients is feasible. Patients in the abdominal approach group can benefit from a shorter operation time and less blood loss. In our small series of varied presentations and tumor extent, we have been able to avoid two-stage surgery, because even short-term interval between the two operations may result in recurrence at the RA.
